# Human and Epstein-Barr Virus miRNA Profiling as Predictive Biomarkers for Endemic Burkitt Lymphoma

**DOI:** 10.3389/fmicb.2017.00501

**Published:** 2017-03-28

**Authors:** Cliff I. Oduor, Mercedeh Movassagh, Yasin Kaymaz, Kiprotich Chelimo, Juliana Otieno, John M. Ong'echa, Ann M. Moormann, Jeffrey A. Bailey

**Affiliations:** ^1^Center for Global Health Research, Kenya Medical Research InstituteKisumu, Kenya; ^2^Department of Biomedical Sciences and Technology, Maseno UniversityMaseno, Kenya; ^3^Program in Bioinformatics and Integrative Biology, University of Massachusetts Medical SchoolWorcester, MA, USA; ^4^Jaramogi Oginga Odinga Teaching and Referral Hospital, Ministry of Medical ServicesKisumu, Kenya; ^5^Program in Molecular Medicine, University of Massachusetts Medical SchoolWorcester, MA, USA; ^6^Division of Transfusion Medicine, Department of Medicine, University of Massachusetts Medical SchoolWorcester, MA, USA

**Keywords:** endemic Burkitt lymphoma, microRNA expression, EBV, RNAseq, miR-10a-5p

## Abstract

Endemic Burkitt lymphoma (eBL) is an aggressive B cell lymphoma and is associated with Epstein-Barr virus (EBV) and *Plasmodium falciparum* malaria co-infections. Central to BL oncogenesis is the over-expression of the MYC proto-oncogene which is caused by a translocation of an Ig enhancer in approximation to the myc gene. While whole genome/transcriptome sequencing methods have been used to define driver mutations and transcriptional dysregulation, microRNA (miRNA) dysregulation and differential expression has yet to be fully characterized. We hypothesized that both human and EBV miRNAs contribute to eBL clinical presentation, disease progression, and poor outcomes. Using sensitive and precise deep sequencing, we identified miRNAs from 17 Kenyan eBL patient tumor samples and delineated the complement of both host and EBV miRNAs. One human miRNA, hsa-miR-10a-5p was found to be differentially expressed (DE), being down-regulated in jaw tumors relative to abdominal and in non-survivors compared to survivors. We also examined EBV miRNAs, which made up 2.7% of the miRNA composition in the eBL samples. However, we did not find any significant associations regarding initial patient outcome or anatomical presentation. Gene ontology analysis and pathway enrichment of previously validated targets of miR-10a-5p suggest that it can promote tumor cell survival as well as aid in evasion of apoptosis. To examine miR-10a-5p regulatory effect on gene expression in eBL, we performed a pairwise correlation coefficient analysis on the expression levels of all its validated targets. We found a significant enrichment of correlated target genes consistent with miR-10a-5p impacting expression. The functions of genes and their correlation fit with multiple target genes impacting tumor resilience. The observed downregulation of miR-10a and associated genes suggests a role for miRNA in eBL patient outcomes and has potential as a predictive biomarker that warrants further investigation.

## Introduction

Endemic Burkitt lymphoma (eBL) is the defining Epstein-Barr Virus (EBV)-associated B cell malignancy in pediatric patients in equatorial Africa, and is characterized by overexpression of the *c-myc* gene, in the vast majority of cases, due to a *t*(8:14) chromosomal translocation (Magrath, 1991; Hecht and Aster, 2000). It has an annual incidence of 5–15 cases in 100,000 children in areas experiencing perennial *Plasmodium falciparum* transmission. Both EBV infection and holoendemic *P. falciparum* are thought to be etiologically linked to the development of this B cell cancer (reviewed in Moormann and Bailey, 2016). eBL is an aggressive lymphoma that can present in a number of different anatomical locations including the jaw, abdomen, orbital area, central nervous system, and breast or a combination of these sites (Mwanda et al., 2004; Ogwang et al., 2008). However, jaw and abdominal tumors are the most common anatomical sites of presentation (50–80% of cases) in pediatric eBL (Magrath, 1991; Buckle et al., 2016).

It has been shown that there are different epidemiological patterns associated with the clinical presentation of eBL. Children with jaw tumors tend to be younger and mostly males, while abdominal BL tumors present more commonly among older children and are equally distributed between males and females (Ogwang et al., 2008; Asito et al., 2010). Differences are also seen in childhood sporadic BL (that is only associated with EBV in 10–20% of cases) where jaw involvement is rare in favor of abdominal and nodal masses (Mbulaiteye et al., 2009). Despite the fact that, eBL has observed clinical and pathologic differences, most studies view eBL as a single clinical entity and attribute survival differences to delayed presentation and variability in treatments (Buckle et al., 2016). Given the epidemiological differences associated with site of tumor presentation that is incorporated into staging disease, there may be molecular differences underlying eBL tumor tropism that have not been fully elucidated although no significant differences in mRNA expression were observed in our recent analysis (Kaymaz et al., 2017).

MicroRNAs (miRNAs) are endogenously expressed, evolutionarily conserved, small single-stranded non-coding RNAs of approximately 18–25 nucleotides in length that fine-tune gene expression (Onnis et al., 2010). In animals, miRNAs control gene expression in a post-transcriptional manner predominantly by partial base-pairing to specific sites located in the 3′ untranslated regions (UTR) of their target mRNAs, triggering degradation, and/or translational inhibition of the target gene (Bushati and Cohen, 2007; Ameres and Zamore, 2013). It is estimated that ~50% of all mammalian protein-coding genes are under miRNA post-transcriptional regulation (Bushati and Cohen, 2007; Ameres and Zamore, 2013). Since their discovery in the nematode in 1993 (Lee et al., 1993), many miRNAs have been identified, and more than 2,000 miRNAs have been described in humans (miRBase release 20; Kozomara and Griffiths-Jones, 2014). Individual miRNAs typically target many transcripts thereby potentiating broad transcriptional control which has conceptually shifted our strategies for targeting oncogenic pathways.

MicroRNAs are involved in many pathological conditions, and their aberrant expression is observed in most cancers (Croce, 2009), including lymphomas and nasopharyngeal carcinoma which is another EBV-associated malignancy (Lee et al., 2016). The pattern of dysregulated miRNA expression can differentiate B cell neoplasms and provided candidate diagnostic biomarkers (Di Lisio et al., 2012). For instance, miR-155 is elevated and highly expressed in Hodgkin lymphoma while low in Burkitt and other non-Hodgkin lymphomas (van den Berg et al., 2003). In BL, miRNA dysregulation has been implicated in c-myc overexpression where hsa-miR-9 may serve as a diagnostic biomarker for identifying BL cases lacking the *c-myc* translocation (Onnis et al., 2010). Studies have also identified candidate prognostic miRNAs in lymphomas including differential expression of miR-21 in diffuse large B cell lymphoma (DLBCL), where its overexpression was associated with poor survival (Li et al., 2015; Zheng et al., 2016). B cell chronic lymphocytic leukemia (CLL) demonstrates down-regulation and deletions of certain miRNAs as well (Balatti et al., 2015). Studies to date, to identify prognostic miRNA biomarkers in eBL are lacking.

EBV is known to drive the proliferation and survival of infected B cells by expressing multiple viral oncogenes (Young and Murray, 2003). Although the virus is present in the vast majority of eBL tumor cells, its viral genes are no longer expressed in order to evade immune surveillance, except for EBNA1 required for replication (Sample et al., 1991). More recently, it has been discovered that the virus expresses numerous viral miRNAs that function to maintain the virus through the subversion of the immune system and protection from apoptosis (Seto et al., 2010; Navari et al., 2014). It has also been demonstrated that EBV miRNAs are also significant viral contributors to the continued survival and proliferation of BL cells where viral loss leading to apoptosis could be compensated by expression of ebv-miR-BHRF1 subset (Vereide et al., 2014; Piccaluga et al., 2016). Combined, these viral miRNA effects may have the potential to worsen prognosis and response to treatment but have yet to be characterized vis-à-vis eBL survival studies and other clinical features.

Of note within the context of eBL, malaria has been shown to induce EBV lytic reactivation (Chêne et al., 2007; Piriou et al., 2009), is associated with higher frequencies of latently infected B cells (Moormann et al., 2005; Mulama et al., 2014; Reynaldi et al., 2016), and has a profound impact on B cell activation which has yet to be fully understood (reviewed in Moormann and Bailey, 2016). To improve our understanding of both human and EBV miRNA within the context of pediatric eBL, we used next generation sequencing, which allows for a more precise delineation and quantification of the miRNA complement, as opposed to hybridization based techniques which are subject to cross hybridization of known miRNAs. We quantified both viral and host miRNAs expressed in clinically annotated eBL jaw and abdominal tumors biopsies collected by fine needle aspirate from children in western Kenya. Apart from the obvious tissue difference between the clinical presentations of eBL, we hypothesized that aberrant miRNA expression may be involved in molecular differences within these tumors, such as amplified metabolic processes that could favor survival or be predictive indicators of poor prognosis. We therefore explored the host and viral miRNA expression activity in eBL tumors comparing their expression in survivors and non-survivors. The aberrant expression patterns of these small regulatory RNAs promise to be rich in biological information that will improve our understanding of the possible influence of miRNAs on eBL patient outcomes.

## Materials and methods

### Sample collection and ethical approval

We collected 17 Fine Needle Aspirates (FNA) of the primary tumors from children aged between 5 and 12 years diagnosed with eBL. The biopsy samples were prospectively collected between 2009 and 2012, prior to chemotherapy treatment at Jaramogi Oginga Odinga Teaching and Referral Hospital (JOOTRH) located in Kisumu City, a regional referral hospital for pediatric cancer cases in western Kenya. Cytology smears were stained using Giemsa/May-Grünwald for morphologic diagnosis. A portion of the biopsy was transferred at the bedside into RNAlater (Qiagen) and stored at −20°C within 3 h. Of the 17 eBL FNA tumor samples used for this study, 7 eBL patients had the jaw tumors while 10 had the abdominal tumors. In support of the diagnosis, RNA-seq expression profile was congruent as all FNA samples showed B cell predominance and high levels of normally associated eBL diagnostic surface markers including, CD19, CD20, CD10, and CD79A/B (Kaymaz et al., 2017). Ethical review and approval for this study was obtained from the Institutional Review Board at the University of Massachusetts Medical School, USA and the Scientific and Ethical Review Unit (SERU) at the Kenya Medical Research Institute (KEMRI) Kenya, and Jaramogi Oginga Odinga Teaching and Referral Hospital (JOOTRH), Kenya Ministry of Health. Children over the age of 7 years were assented. Parents and legal guardians of the study participants provided written informed consent.

### Small RNA isolation

Small RNA along with large RNA and DNA was isolated from FNA primary biopsy tissues stored in RNAlater using the AllPrep DNA/RNA/protein mini kit according to manufacturer's instructions (Qiagen). Small RNA abundance and integrity were determined after isolation using a Nanodrop spectrophotometer (Thermo Fisher Scientific, Waltham, Massachusetts, USA) and an Agilent Bioanalyzer (Agilent Technologies, Santa Clara, CA), respectively. Only samples of small RNA with a miRNA concentration >10 ng/μl and RIN (RNA integrity number) >8.0 were considered for small RNA library preparations.

### Preparation of microRNA libraries for deep sequencing

Seventeen indexed miRNA libraries were prepared using the Illumina Truseq Small RNA Library Preparation Kit (Illumina Inc., San Diego, CA, USA). Procedure for the preparation of the sequencing libraries were performed according to the manufacturer's protocol. Briefly, a 3′-adaptor sequence was first added to the 3′-end of the small RNA molecule then a 5′ adaptor sequence was added to the 5′-end of the small RNA. The adaptor ligated RNA was then reverse transcribed into cDNA, which was eliminated by adding RNase. The libraries were separated from adapter dimers by size fractionation in 8% TBE polyacrylamide gel (Life Technologies, Carlsbad, CA, USA). The 150 bp small RNA libraries were excised and purified from the gel. We then used the Agilent High Sensitivity DNA Kit (Agilent Technologies, Colorado Springs, CO, USA) to quantify the molarity and confirm the size distribution. The 17 Indexed miRNA libraries were pooled in equimolar concentrations and sequenced on one lane of an Illumina HiSeq 2000 platform (Illumina Inc., San Diego, CA, USA). The fastq files were produced using the CASAVA pipeline v2.0 (Illumina Inc., San Diego, CA, USA) and all generated sequence were deposited in NCBI dbGAP (accession number: phs001282.v2).

### Bioinformatic analysis pipeline and miRNA expression profile generation

Preliminary quality control analysis of the 17 fastq files was carried out with FASTQC software v0.10.0 (Andrews, 2014). Cutadapt v1.1 (Martin, 2011) was then used to trim off the 3′-adaptor sequence from the sequencing reads. Novobarcode (Novocraft)^1^ was used to de-multiplex the samples based on the 6-nucleotide barcode that was added to the small RNA sequencing library of each sample. Reads shorter than 17 nucleotides after adaptor trimming and barcode removal were discarded. The trimmed reads were then further checked for the presence of any artificial sequences from the adaptor or barcode using fastqc. Reads passing all the above filters, including filtering out ribosomal RNAs, were aligned to the human genome (hg19) using bowtie (Langmead, 2010). Reads were than aligned to a concatenated wild type reference genome of EBV and human [RefSeq ID: NC_007605, GRCh37 (hg19), respectively]. Reads that did not uniquely align to the EBV genome were discarded to insure unique identity of the EBV miRNA reads. The resulting sequences were subjected to our computational pipeline (Figure S1), which consists of a number of in-house scripts followed by miRDeep2 (Friedländer et al., 2012) analysis to determine the miRNA counts for each of the eBL samples. Briefly, we used the mapper module in miRDeep2 (default settings) to preprocess the sequencing reads and map to the reference human or EBV genome producing an arf file, however to identify the viral miRNAs we included a −f flag to obtain maximum target detection for the low level viral miRNAs. The miRDeep2 quantifier module (default settings) was then used to determine the expression counts of both the human and viral miRNAs.

### Differential miRNA expression analysis

Expression analysis of miRNA-Seq data was performed with the R/Bioconductor version 3.0 package *EdgeR* (v2.4.6) (Robinson et al., 2010), which is designed for use with digital gene expression data. Count numbers of each miRNA were imported to *EdgeR*, log2 transformed, and normalized based on negative binomial distribution model to account for both technical and biological variability. Human and EBV miRNAs were analyzed as one group to control conservatively for multiple comparisons. The miRNA expression counts normalization involved estimating the sample-specific normalization factors to rescale the observed counts using the TMM (trimmed mean of *M*-values) method (Supplementary Data Sheet 1). Only miRNAs that had at least 10 counts per million (cpm) reads in all the samples were analyzed for evidence of differential miRNA expression. This minimal level helps to ensure that expression difference are not simply low level statistical noise and of likely biologic consequence (Kozomara and Griffiths-Jones, 2014). Within edgeR, *p*-values were adjusted for multiple testing with the Benjamini and Hochberg (1995) approach for adjusting the false discovery rate (FDR).

### Validation of miRNA expression using qRT-PCR

MiRNA expression levels were validated by quantitative reverse transcription polymerase chain reaction (qRT-PCR) using TaqMan miRNA assay protocol on the BioRad CFX96 Real-Time System. The experiments were run in triplicates on the 17 miRNA samples sequenced. To normalize the expression levels of the target miRNA by correcting for the amount of cDNA loaded to the PCR reaction, we used the comparative Ct method. All Ct-values were normalized to an endogenous control (U54), and ΔCt-values were calculated, where ΔCt = Ct (miRNA) − Ct (U54) (Livak and Schmittgen, 2001; Marabita et al., 2016). Relative expression values (2^−ΔCt^) were plotted and compared. To verify mean differences among the groups, the normalized PCR data was analyzed using the wilcoxon rank test in R. A two-sided *p* < 0.05 was considered statistically significant.

### Functional and pathway enrichment analysis of the validated target genes controlled by the differentially expressed miRNAs

Since the capacity of miRNA to limit gene expression can result in functional and phenotypic consequences, we identified the genes controlled by the aberrantly expressed miRNAs between the jaw and abdominal eBL tumors. The entrez gene IDs of the target genes which were controlled by the miRs were imported into the functional Annotation tool of Database for Annotation, Visualization and Integrated Discovery (DAVID) version 6.7 (Dennis et al., 2003; Huang et al., 2007). DAVID was used to provide biological functional interpretation of the validated targets of the DE miRNA and identifies the most relevant KEGG pathway and gene ontology (GO) categories, composed of the genes enriched in the given set, followed by an output of statistical significance evaluated by the modified Fisher's exact test *p*-values [and corresponding false discovery rate (FDR)], which were calculated to identify which biological pathways are significantly enriched in the miRNA target list.

### miRNA target gene identification and correlation coefficient calculation

miRNAs regulate expression of specific genes via hybridization to mRNA transcripts to promote RNA degradation, inhibit translation, or both (Behm-Ansmant et al., 2006). To investigate the biological relevance of the identified miRNA, we identified all the validated target genes for the DE miRNA using the Validated Target module of the miRWalk2.0 (Dweep et al., 2011; Dweep and Gretz, 2015) database. Using mRNA expression data from a published RNAseq dataset (Kaymaz et al., 2017, accession number: phs001282.v1) for the eBL patients in this study, pearson correlation coefficients between a miRNA and its validated target mRNAs were computed using R statistical programming language, to determine whether the expression levels of each miRNA and of its mRNA targets show any correlation. Bootstrap *p*-values were calculated with replacement from 10,000 replicates sampled from all expressed mRNAs.

## Results

### Clinical information and sequencing of the eBL samples

To investigate the composition of miRNAs in eBL, we sequenced small RNAs from 17 primary tumor FNA biopsies collected from Kenyan children with median age 6.8 years old (Table 1). Of the eBL patients included in this study, 41% (7/17) presented predominantly with a unilateral jaw tumor whereas 59% (10/17) had abdominal masses with no apparent tumors above the diaphragm. In terms of patient outcomes, two patients died soon after admission to hospital and prior to receiving any chemotherapy, seven patients died during the in-hospital course of chemotherapy, and five patients completed induction-consolidation therapy and were discharged home. One patient was admitted with relapse having been previously treated while two patients were lost to follow-up because they were transferred to another hospital, therefore their outcomes are unknown (Table 1). For each of the 17 samples, we generated an average of 1.4 million reads per library and 2,042 distinct human miRNAs and 43 distinct viral miRNAs were detected (Figure S2, Table S2).

**Table 1:** **Clinical and demographic characteristics of eBL patients**.

**Characteristics**	**Total (*N* = 17)**
Age (years), median (range)	7.5 (5–12)
**GENDER**, ***N*** **(%)**	
Male	11 (64.7%)
Female	6 (35.3%)
**TUMOR FNA SITE**, ***N*** **(%)**	
Jaw	7 (41%)
Abdomen	10 (59%)
**IN-HOSPITAL SURVIVAL-STATUS**, ***N*** **(%)**	
Survived^¥^	5 (29.4%)
Died^¶^	7 (41.2%)
Relapsed	1 (5.9%)
Died prior to chemotherapy^§^	2 (11.8%)
Unknown outcome (patient referred to another hospital)	2 (11.8%)

¥ *eBL patients who completed chemotherapy, were discharged from hospital and still alive after 2 years of follow-up*.

¶ *Patients who had started chemotherapy but died during the course of treatment*.

§ *eBL patient who died before starting chemotherapy*.

### miRNAs show similar pattern of expression without tumor subtypes by hierarchical clustering

**Figure 1:**
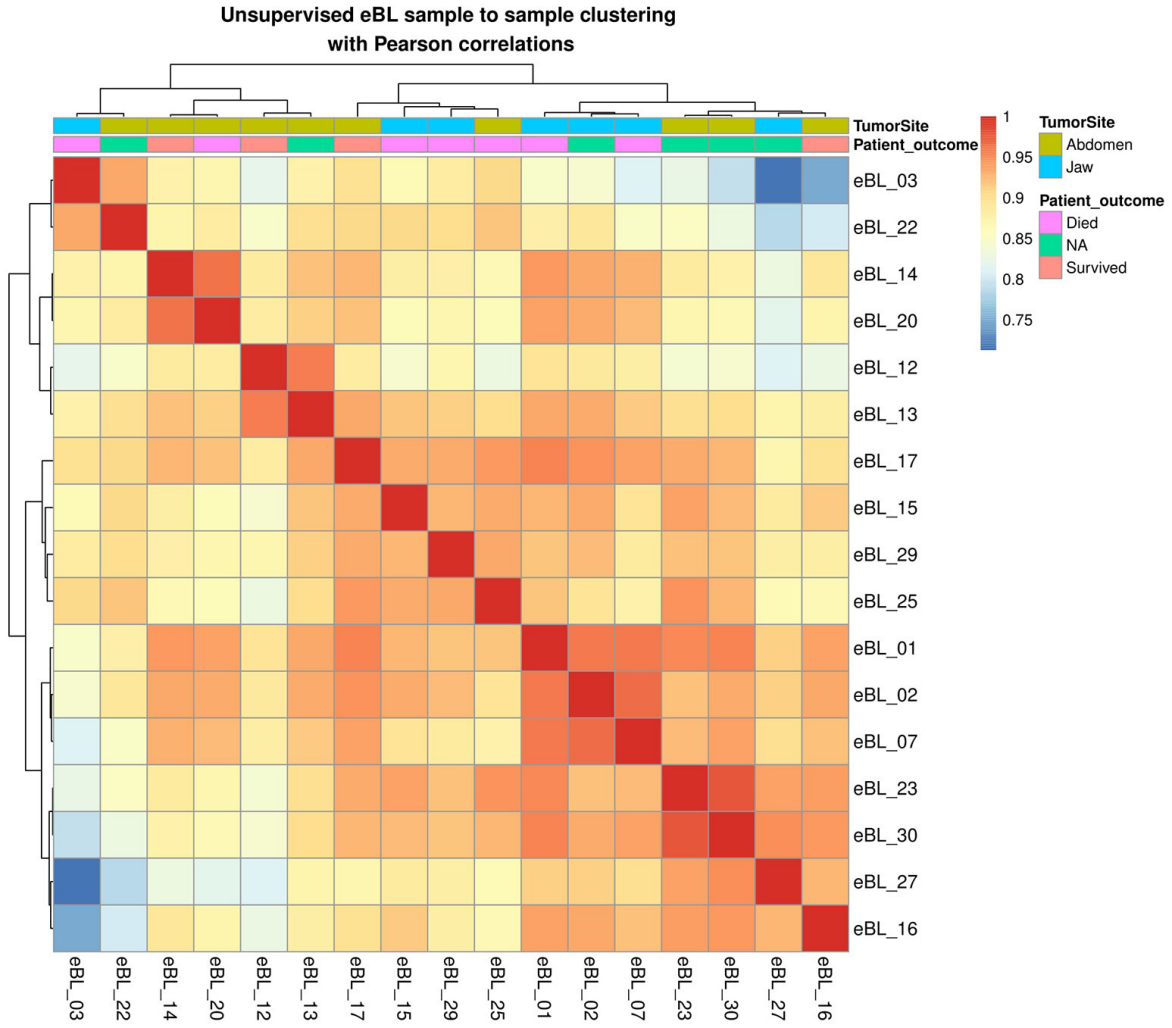
**Sample to sample hierarchal clustering of BL tumors based on miRNA expression profiles with highest correlation of variation (CV)-values (calculated using regularized log transformed all miRNA expression)**.

To evaluate whether tumor miRNAs expression patterns were suggestive of the existence of eBL subtypes, we performed unsupervised hierarchical clustering of the expression of all miRNAs. The overall correlations among eBL samples were extremely high (*r* > 0.9, average; Figure 1). The most disparate eBL samples (eBL_03 and eBL_22 compared to eBL_27 and eBL_16) still show a high degree of correlation (*r* = 0.7–0.8). There was no discernible clustering based on tumor site designation or in-hospital survival. Similarly, principal component analysis and multidimensional scaling showed no discernible separation based on tumor presentation site (Figure S3). Overall, this suggests that eBL tumors are relatively homogeneous without overt subtypes nor altered miRNA expression signatures based on tumor presentation site consistent with lack of subgroups examining mRNA expression (Kaymaz et al., 2017).

### Differentially expressed (DE) miRNAs relative to tumor presentation site and in-hospital survival

**Figure 2:**
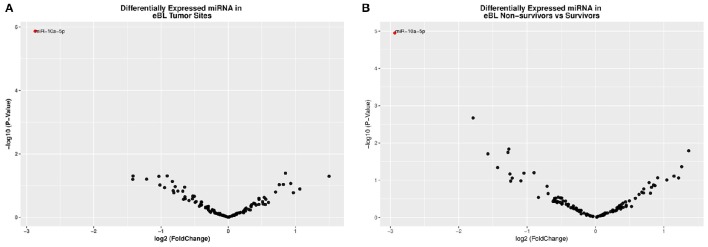
**(A)** A volcano plot displaying the statistically significant (*p* < 0.01 and FDR < 0.1) results and shows the relationship between the significance of miRNAs detected and the fold-change between eBL jaw and abdominal tumors. miR-10a-5p showed 2.7-fold lower abundance in the jaw vs. abdominal tumors. The red colored circles represent miR-10a-5p which is differentially expressed with *p* < 0.01 and FDR < 0.02. The down-regulated miRNAs are signified by a negative fold-change value and *vice versa*. **(B)** A volcano plot representing the significance of miRNAs (−log of the *p*-value) vs. the fold change difference in eBL Non-survivors' vs. Survivors. Hsa-miR-10a-5p was also downregulated in the eBL non-survivors.

Given eBL tumors showed no distinctive clusters of overall miRNA expression patterns, we focused our analysis on the differential expression of individual miRNAs. When the expression of both human and viral miRNAs was compared between jaw and abdominal eBL tumor samples, one miRNA, hsa-miR-10a-5p (log2FC = −2.873, *p* = 0.0018 and FDR = 7.67e-06), was found to be significantly DE (Table 2; Table S3). This miRNA showed lower expression in eBL jaw tumors compared to abdominal tumors (Figure 2A).

**Table 2:** **Differentially expressed miRNA in eBL jaw tumors compared to abdominal tumors**.

**miRNA**	**Accession**	***P*-value**	**False discovery rate (FDR)**	**Fold change (FC) log2FC**	**Jaw tumor vs. abdominal tumor**
hsa-miR-10a-5p	MIMAT0000253	7.67e-06	0.001857	−2.873	Down-regulated

Examining differential expression with regards to in-hospital survival, we compared the miRNA expression profiles of patients who died during the course of treatment to patients who successfully completed chemotherapy and were discharged (i.e., in-hospital survivors). Our analysis detected only one significant DE miRNA, hsa-miR-10a-5p (log2FC = −2.935, *p* = 1.12e-05 and FDR = 0.0013) (Table 3), which was significantly lower in non-survivors relative to survivors (Figure 2B; Table S3).

**Table 3:** **Differentially expressed miRNA in eBL non-survivors compared to survivors**.

**miRNA**	**Accession**	***P*-value**	**False discovery rate (FDR)**	**Fold change (FC) log2FC**	**Non-survivors vs. survivors**
hsa-miR-10a-5p	MIMAT0000253	1.12e-05	0.0013	−2.935	Down-regulated

From our miRNA sequencing data we observed the expression of 43 known ebv miRNAs in the eBL tumor cells representing on average 2.7% with a wide range from 0.01 to 11.9% of all miRNA expression (Tables S1, S2). None of the viral miRNAs demonstrated significant association with eBL anatomical tumor presentation site or patient outcome.

### miR-10a-5p validation by qRT-PCR

**Figure 3:**
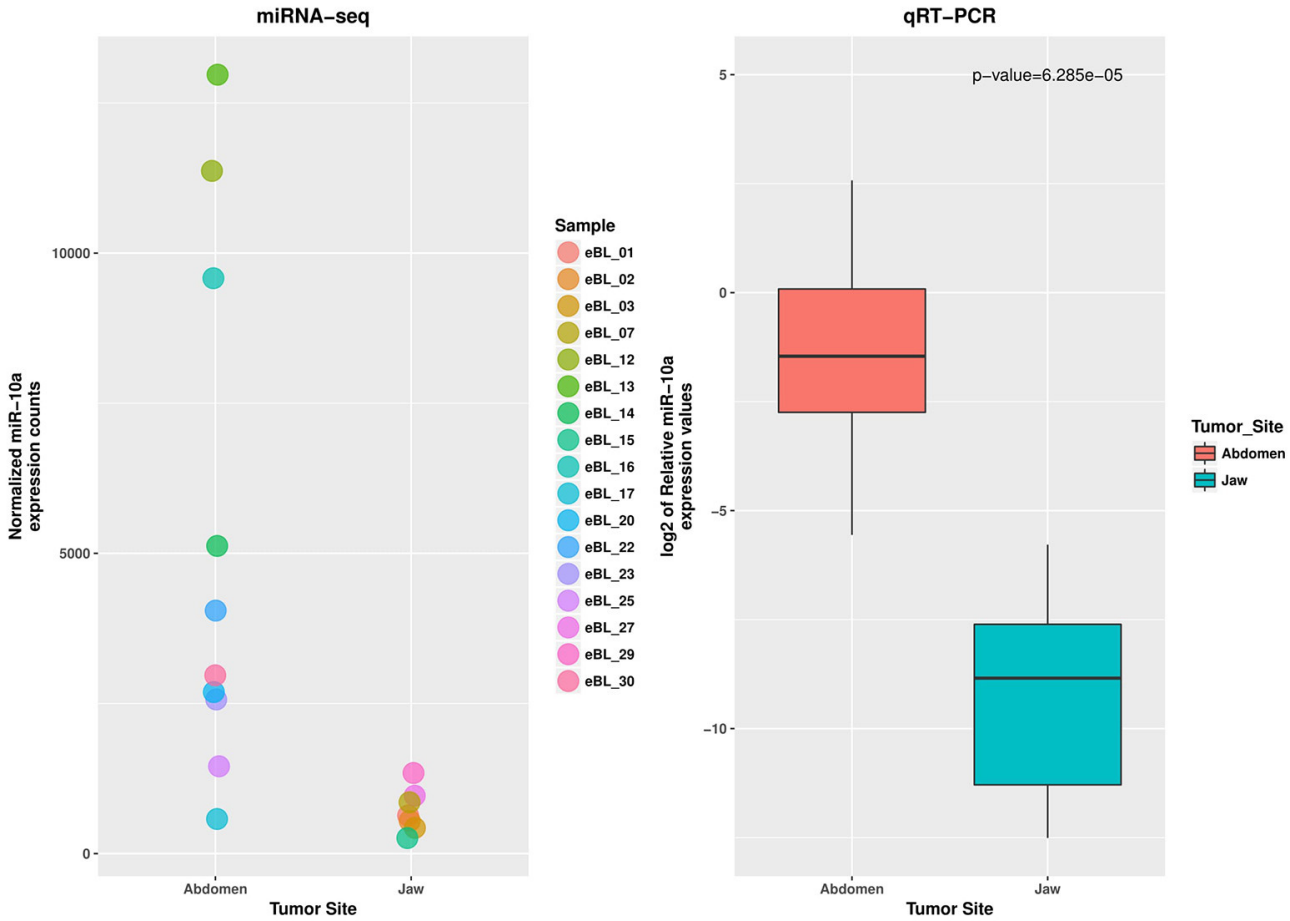
**qRT-PCR validation of miR-10a-5p expression difference in jaw vs. abdominal tumors**. The jitter plot shows the normalized expression values from the miRNA sequencing experiment. The box plot show the log2 median expression levels of miR-10a-5p, confirmed as downregulated in Jaw tumors, estimated in terms of normalized fluorescence intensity.

**Figure 4:**
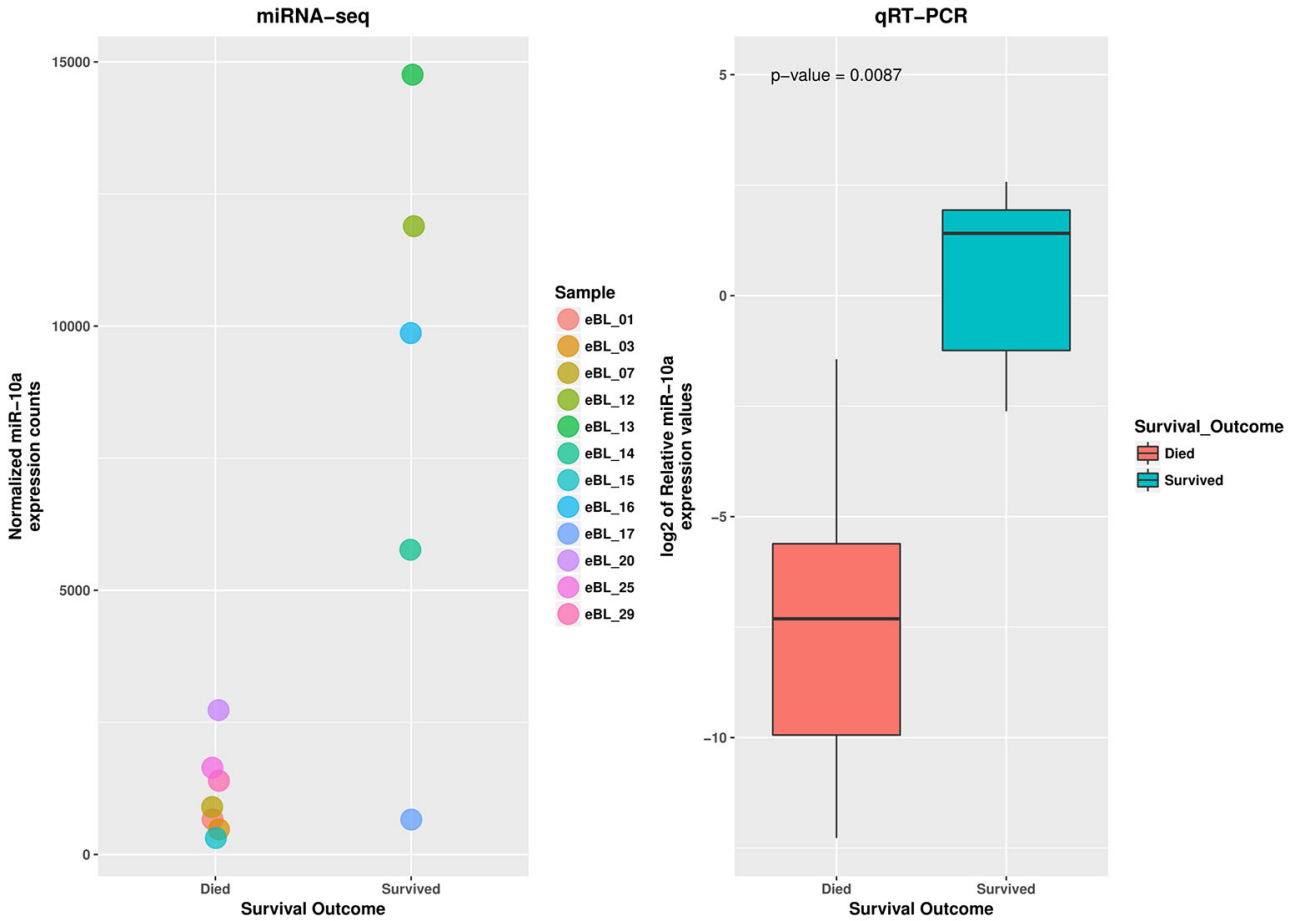
**qRT-PCR validation of miR-10a-5p expression difference in eBL survivors vs. non-survivor tumor samples**. The jitter plot shows the normalized expression values from the miRNA sequencing experiment. The box plot shows the log2 median expression levels of miR-10a-5p, confirmed as downregulated in eBL non-survivor patients, estimated in terms of normalized fluorescence intensity.

To assess the reproducibility and validity of the miR-10a expression levels by deep sequencing we measured its levels by qRT-PCR. The wilcoxon rank test confirmed the difference in expression level for miR-10a-5p between the jaw and abdominal tumors, and also between eBL survivors and non-survivors (*p* = 6.29e-05 and 0.0111, respectively). The boxplot in Figures 3, 4, shows the median distribution levels of the log2 normalized relative quantity of miR-10a-5p in the eBL tumors. The qRT-PCR results showed good correlation with sequencing (*r* = 0.77) based on a correlation test between qRT-PCR miR-10a expression levels and miR-10a expression levels from the sequencing experiment. Overall, this further validates our sequencing results (Figure S4).

### Gene ontogeny and functional enrichment of validated targets

To investigate the potential functional significance of the DE miRNA (miR-10a-5p), its validated target genes were identified from Tarbase (Sethupathy et al., 2006) and miRTarbase (Hsu et al., 2014) in the validated target module of miRWalk2.0 (Table S3). A total of 437 genes were identified to be targeted by miR-10a-5p. Of these 251 targets showed appreciable levels of expression in our samples with a median expression of >10 cpm.

Gene ontology (GO) function and KEGG pathway enrichment were performed by mapping the validated target genes of the DE miRNA to the GO and KEGG database, respectively, using DAVID (Figures S6, Figure 6). For the two DE miRNAs between jaw and abdominal tumors, Several KEGG pathways were identified as significantly enriched (*p* < 0.05; Figure 6). The target genes of the down-regulated miR-10a-5p in eBL non-survivors and jaw tumors, were significantly enriched in pathways of cancer (hsa05200) *p* = 0.014, focal adhesion signaling pathway (hsa04510) *p* = 0.013, and EBV infection pathway (hsa05169) *p* = 0.018.

### miRNA target gene identification and correlation of expression profiles between miR-10a-5p and its validated target genes

**Figure 5:**
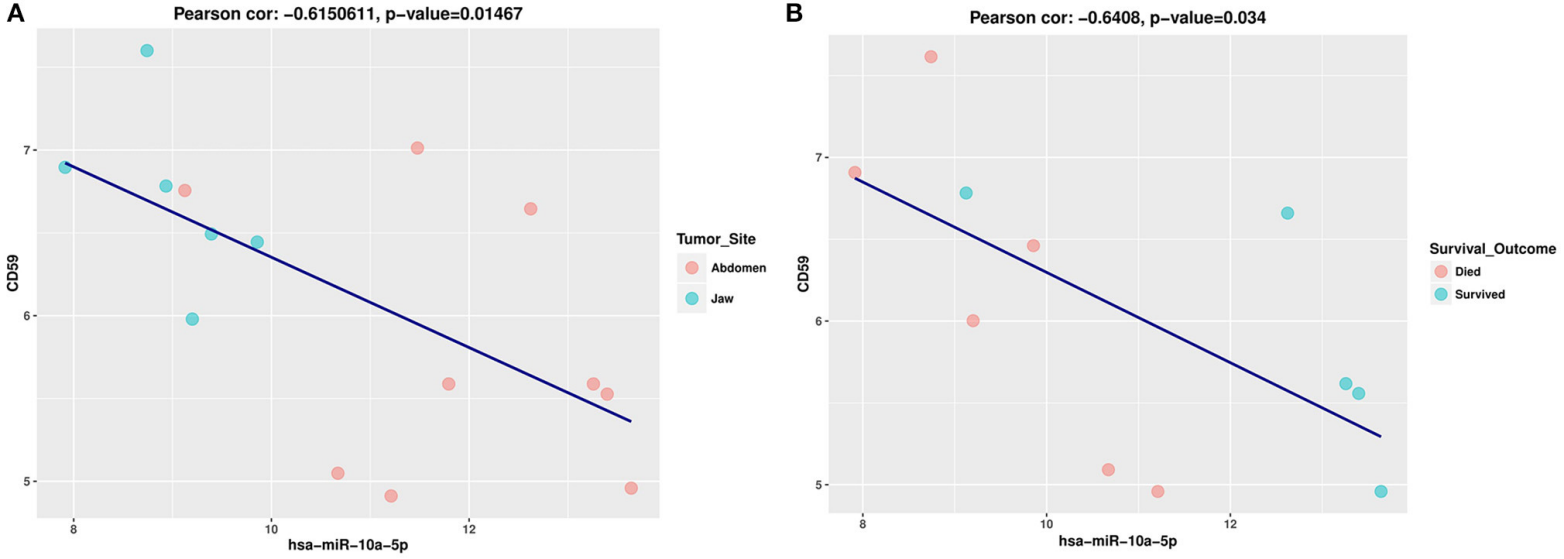
**(A)** Pearson correlation scatter plot showing a negative correlation between hsa-miR-10a-5p and CD59 expression levels in eBL patients. The horizontal and vertical axis represents the log2-expression values of the miRNA and mRNA, respectively. These plots show the relationship between hsa-miR-10a-5p expression and CD59 expression levels in eBL patients based tumor site. **(B)** Pearson correlation scatter plot showing a negative correlation between hsa-miR-10a-5p and CD59 expression levels in eBL patients based on patient survival outcome. The horizontal and vertical axis represents the log2-expression values of the miRNA and mRNA, respectively. The plots show the relationship between hsa-miR-10a-5p expression and CD59 expression levels in eBL patients based on patient outcome.

In general target transcript levels, should be impacted by changes in miRNA levels. Previous miRNA and mRNA target analysis have noted that the majority of target gene expression do not show simple negative correlation but rather the most predominant finding is increased rates of both positive and negative correlation (Ruike et al., 2008; Wang and Li, 2009). The role of positive correlations due to positive feed-forward loops have been increasingly recognized (Friard et al., 2010; Chen et al., 2011; Zhang et al., 2015). We performed a pairwise correlation coefficient analysis between miR-10a-5p expression levels and the expression levels of its 251 validated target genes with a median count >10 cpm from an RNAseq data set for the eBL patients. We found five positive and three negative correlations *p* < 0.05, which overall, was not significant based on bootstrapping replicates of random genes. Given the levels of miR-10a are very low in a number of samples we examined the upper half (log2miR-10a levels >10) and lower half (log2miR-10a levels <10) separately to look for regulation at higher levels of the miRNAs. In this case, we saw more numerous correlations when the gene was more highly expressed, 22 genes with significant correlations (*P* < 0.05), which was more than expected by chance (*p* = 0.0352) based on a simulation test on random gene selection. The lower half showed poor correlation suggesting that at low levels there is poor regulation and other factors predominant in determining target levels. Of the validated target gene set, there are multiple genes with some degree of correlation that would explain miR-10a association with patient survival outcome by impairing apoptotic death, CD59, API5, MDM4, and YY1 showed an inverse relationship with miR-10a levels. Increased levels of each of these genes could impair apoptotic death and influence eBL patient prognosis. Positively correlated genes that would decrease and increase apoptotic death of the tumor cells include BCL2L13, and PTEN. While only CD59 showed a significant (*P* < 0.05) inverse correlation with miR-10a levels (Figures 5A,B), overall, this analysis suggests that miRNA is modulating multiple genes in a consistent way that could impair patient survival.

**Figure 6:**
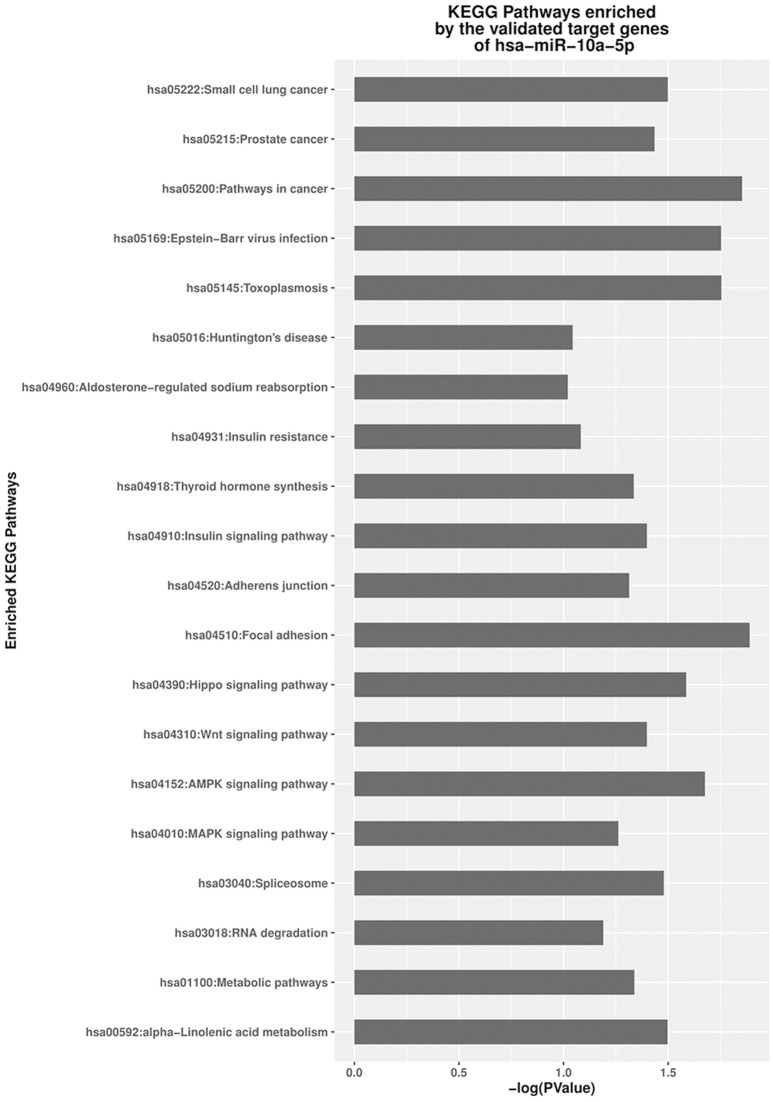
**Significantly enriched signaling pathways of the validated targets of miR-10a-5p**.

## Discussion

Previous studies, have shown that cancer tumors with different anatomical sites have unique patterns of miRNA expression (Lu et al., 2005; Petillo et al., 2009). Using next generation sequencing and controlling for multiple comparisons, we identified miR-10a-5p to be DE between the two major eBL tumor presentation sites, and also uncovered pathways that are possibly enriched by this DE miRNA. miR-10a-5p expression was also found to be significantly lower in eBL patients who did not survive after starting chemotherapy treatment. This miRNA was downregulated in eBL patients with the jaw tumor and also in eBL non-survivors with >2.8- and 2.9-fold decrease, respectively. To gain some insight into the biological functions deregulated by miR-10a-5p, we identified its validated targets and used mRNA expression data to add biological significance by performing a pairwise correlation analysis. We show that correlated transcription suggests that these genes likely work together to inhibit apoptosis and minimize sensitivity to chemotherapy.

In support of miR-10a-5p role in cancer, downregulation of miR-10a-5p has also been implicated in other cancer studies (Jansson and Lund, 2012). Downregulation has been reported in chronic myeloid leukemia (CML) and associated with increased cell growth of the CML cells (Agirre et al., 2008). Experimental re-expression of hsa-miR-10a-5p in CML cells decreased cell growth, thus supporting the functional role of hsa-miR-10a in CML disease progression (Agirre et al., 2008).

The potential relevance of the miR-10a-5p in eBL tumors can best be discussed by examining its targets and implications for altering their function. Since the expression of the observed DE miRNA is downregulated in jaw compared to abdominal tumors and is associated with poor survival, the dysregulation of its target genes could lead to significant biologic differences that may influence patient survival (Yousef and Allmer, 2014). Our analysis supports that miR-10a-5p is biologically relevant and that there is a set of targets that potentiate tumor resistance to apoptosis and chemotherapy. MiR-10a-5p downregulation in the jaw tumors could lead to hyperactivation of its target CD59 (Helwak et al., 2013), a potent inhibitor of the complement membrane attack complex. Experimental evidence has shown that CD59 is effective at protecting cancer cells from antibody (i.e., rituximab) mediated complement-dependent cytotoxicity (You et al., 2011). High expression of CD59 has also been correlated with poor overall survival and progression free survival in lymphoma patients treated with R-CHOP (rituximab, cyclophosphamide, doxorubicin, vincristine, and prednisone; Song et al., 2014). Lack of post-transcriptional regulation of CD59 due to miR-10a-5p downregulation in eBL patients would enhance tumor cell survival and possibly increase relapse rates. We show a significant inverse correlation between miR-10a-5p and CD59 expression levels in eBL tumors (Figures 5A,B), pointing to a potentially functional miRNA-mRNA relationship which could promote tumor cell survival, chemoresistance and poor outcomes.

Considering the aggressive nature of the defects leading to eBL development, downregulated miR-10a-5p could also promote the hyperactivation of API5 (apoptosis inhibitor 5), an apoptosis inhibitory protein, which renders tumor cells resistant to T cell initiated apoptosis (Noh et al., 2014). API5 has been shown to impair the cytotoxic effect induced by chemotherapeutic drugs (Faye and Poyet, 2010), while miR-10a-5p confers post-transcriptional regulation of API5 gene (Karginov and Hannon, 2013). An inverse correlation was observed between API5 and miR-10a-5p level, suggesting this interaction may influence response to treatment and eBL patient survival. We also observed an inverse relation between miR-10a and MDM4 levels. Given that miR-10a-5p targets MDM4 and MDM2, inhibitors of p53 (Francoz et al., 2006), this interaction could influence survival of eBL patients (Leroy et al., 2002; Kishore et al., 2011; Yousef and Allmer, 2014).

Molecular network analysis enables us to characterize the most relevant pathways involved in the miRNA targets *in silico* (Murray et al., 2010). Human miRNA targets regulated by an individual miRNA generally constitutes function-associated molecules. It is therefore plausible that even a small change in the expression of a single miRNA could affect a wide range of signaling pathways and networks involved in diverse biological functions (Murray et al., 2010). Through the enrichment and function analysis using DAVID database, we propose that the target genes of the DE miRNA in the jaw tumor and non-survivors, may lead to the enhancement of many important biological processes, including positive regulation of cell proliferation (Figures S5, S6). Enrichment analysis indicated that the target genes of the identified DE-miRNA were mainly involved in cancer-related KEGG pathways, including, focal adhesion signaling, and pathways in cancer which were significantly enriched as the top most relevant KEGG pathways. These appear to impact a survival advantage for tumor cells. FAK (Focal adhesion kinase) signaling has been associated/related to transformation, metastasis, migration, and poor outcome (Miyazaki et al., 2003; Benlimame et al., 2005; Natarajan et al., 2006) in many solid tumors and also in hematologic malignancies. This pathway is also presumed to inhibit signaling pathways leading to the activation of caspase 3 (Sonoda et al., 2000). FAK pathway enrichment by the targets of miR-10a-5p, as a result of the upregulation of these genes, would promote apoptotic escape of thus sustaining tumor cell survival or increased tumor burden. This pathway enrichment analysis provides insight into the molecular events that may lead to poor outcome among eBL patients.

Our observation that eBL tumors express varying levels of EBV miRNAs suggested a role for EBV non-coding transcripts in eBL disease progression, as postulated by another group (Piccaluga et al., 2016). The BHRF1 miRNA cluster has been shown to strongly potentiate the transforming properties of EBV (Li et al., 2012; Ma et al., 2016), and have been shown to downregulate genes that antagonize or prevent B cell growth (Bernhardt et al., 2016). However, based on our study of Kenyan eBL patients we were not able to demonstrate any impact of EBV miRNA on patient outcome or anatomical location of tumor. It is possible that viral miRNA are important during B cell transformation but no longer informative after tumorigenesis.

A limitation of this study is sample size. However, 17 eBL tumors is comparable to other published studies of miRNA associations with diagnosis or outcome (Sun et al., 2004). Nevertheless, the power to detect nuanced differences is modest and there may be biologically meaningful differences in miRNA expression that we have not detected. Stochastic noise may have influenced our findings although we carefully controlled for significance and false discovery rate. However, larger, multi-site sample sets are needed to truly validate the functional role of host and viral miRNA in eBL. The role malaria plays, either directly or indirectly (Moormann and Bailey, 2016), in aberrant miRNA expression was not addressed within our study but warrants investigation. The dynamic mechanisms by which miRNA target genes and influence cancer progression is clearly an active area of investigation.

In conclusion, we found evidence for an altered miR-10a-5p expression pattern between different tumor sites. We show that there is low expression of miR-10a-5p in eBL patients presenting with jaw tumors and overall in patients who died. A significant inverse correlation was observed between miR-10a-5p and CD59 expression, implying a biologically relevant, functional miRNA-mRNA target interaction, which would enhance tumor cell survival and thus render the tumor less sensitive to chemotherapy. These findings provide novel insight into the role of miRNA in the pathogenesis of eBL and a basis for future research on miR-10a-5p and CD59 involvement in eBL patient outcomes. Understanding how miRNA influence tumor progression has implications for the development of novel therapeutic interventions to improve outcomes for children diagnosed with eBL.

## Author contributions

Conception and design of the study: CO, KC, AM, JB. Acquisition of samples: CO, JO, JMO, AM. Performed the experiments: CO, YK, MM. Analysis and interpretation of data (e.g., statistical analysis, biostatistics, computational analysis): CO, MM, YK, AM, JB. Writing, review, and/or revision of the manuscript: CO, MM, YK, KC, JO, JMO, AM, JB.

## Funding

This study was supported by the US National Institutes of Health, National Cancer Institute R01CA134051, R01CA189806 (AM) and The Thrasher Research Fund 02833-7 (AM), UMCCTS Pilot Project Program U1 LTR000161-04.

### Conflict of interest statement

The authors declare that the research was conducted in the absence of any commercial or financial relationships that could be construed as a potential conflict of interest.

## Footnotes

^1^Available online at: http://www.novocraft.com/ (Accessed November 3, 2016).

## Supplementary material

Supplementary Data Sheet 1 **Normalized expression counts of Human and EBV miRNAs expressed in eBL tumor**.

Figure S1 **Bioinformatic workflow for host and viral miRNA analysis pipeline (processed raw reads and identified counts of known human and ebv miRNAs in high-throughput sequencing data)**.

Figure S2 **Hierarchical clustering of all the host and viral miRNAs expressed in the eBL tumor cells**.

Figure S3 **PCA plots of eBL tumors sites**. The sample names were colored per their groups (red represents the jaw tumors and black represented the abdominal tumors). The tumor samples don't show well-defined grouping based on tumor site in the PCA analysis.

Figure S4 **Pearson correlation scatter plots showing a positive correlation between qRT-PCR miR-10a-5p expression levels and miRNA-seq miR-10a-5p expression levels**.

Figure S5 **Pearson correlation scatter plots showing the relationship between hsa-miR-10a-5p expression and its target gene expression in eBL patients**. The blue points represent the eBL patients with the jaw tumors and the red points represents eBL patients with the abdominal tumors.

Figure S6 **Gene ontologies enriched (Biological processes, Cellular components and Molecular functions) by the validated target genes of miR-10a-5p**.

Table S1 **Key patient characteristics and small RNA sequence mapping information**.

Table S2 **miRNA differential expression analysis results**.

Table S3 **Validated target genes of miR-10a-5p and miRNA-target mRNA expression correlation coefficients**.
