# Supplementary figures and images for: Human and Epstein-Barr Virus miRNA Profiling as Predictive Biomarkers for Endemic Burkitt Lymphoma
Source: Front Microbiol. 2017 Mar 28;8:501. doi: 10.3389/fmicb.2017.00501 (PMC5368269; doi:10.3389/fmicb.2017.00501)

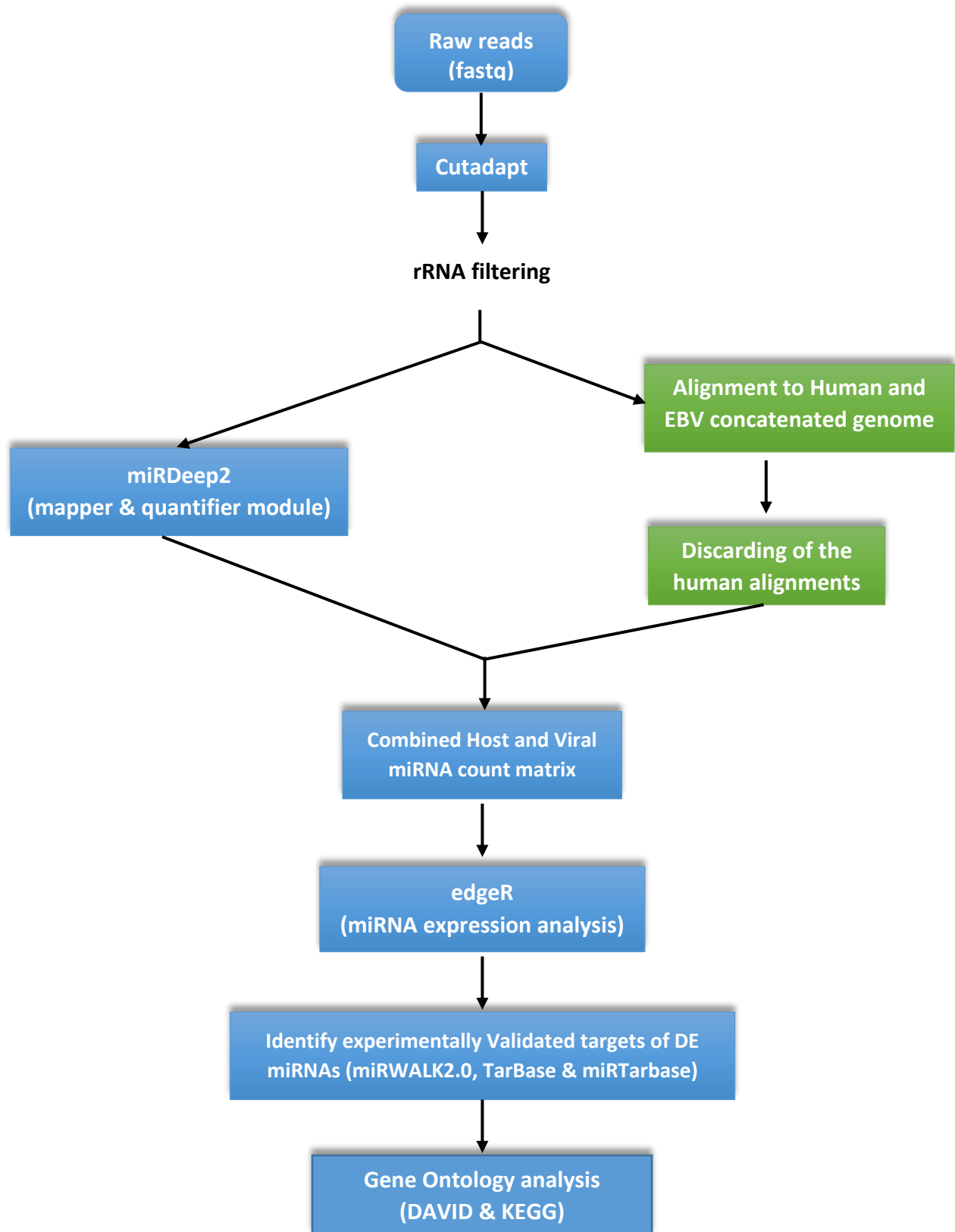

Supplement: Figure S1 — Bioinformatic workflow for host and viral miRNA analysis pipeline (processed raw reads and identified counts of known human and ebv miRNAs in high-throughput sequencing data). [file Image1.PDF]

Expressed host and viral miR  
in eBL tumors

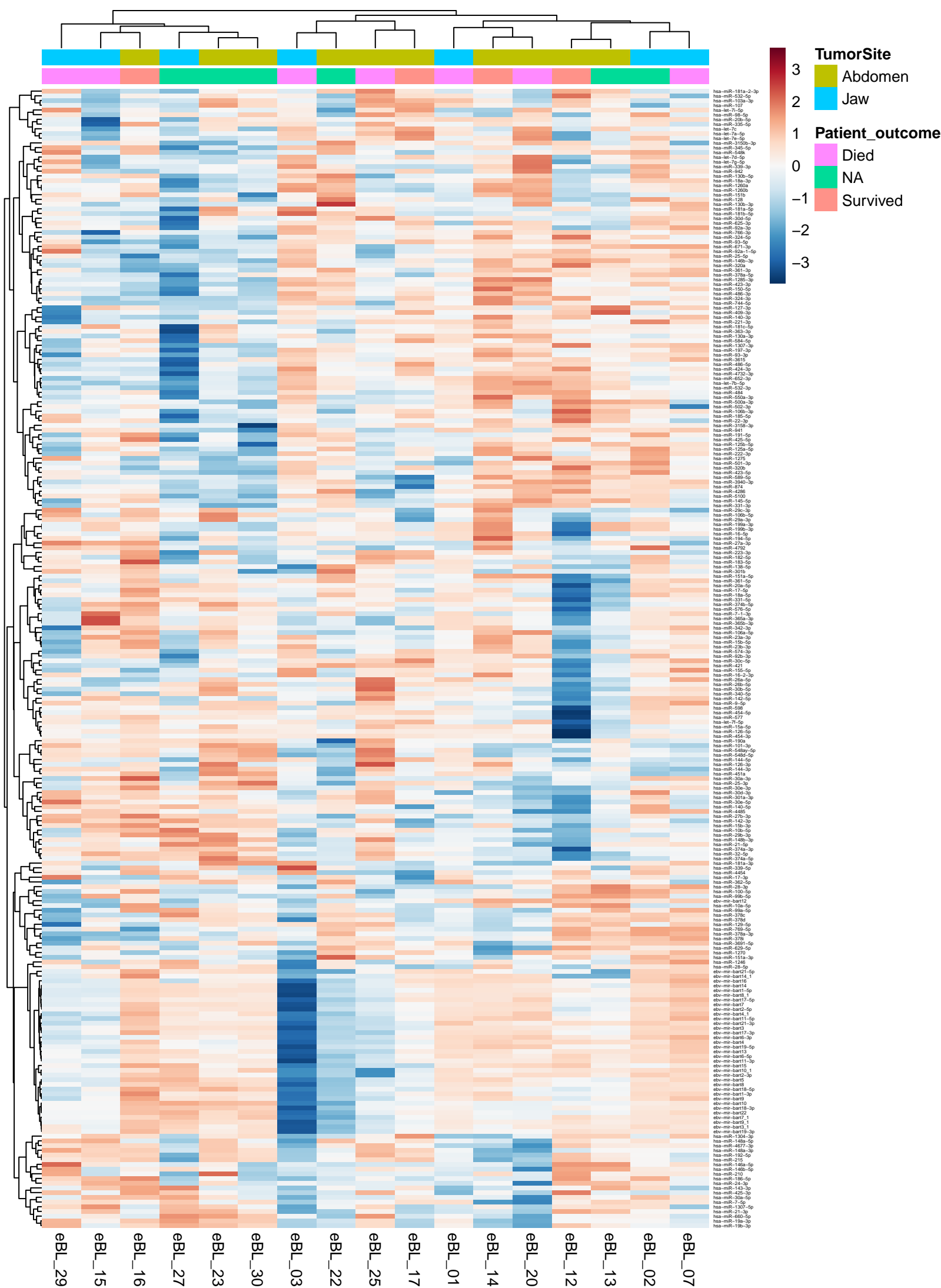

Supplement: Figure S2 — Hierarchical clustering of all the host and viral miRNAs expressed in the eBL tumor cells. [file Image2.PDF]

PCA Plot of eBL tumor samples

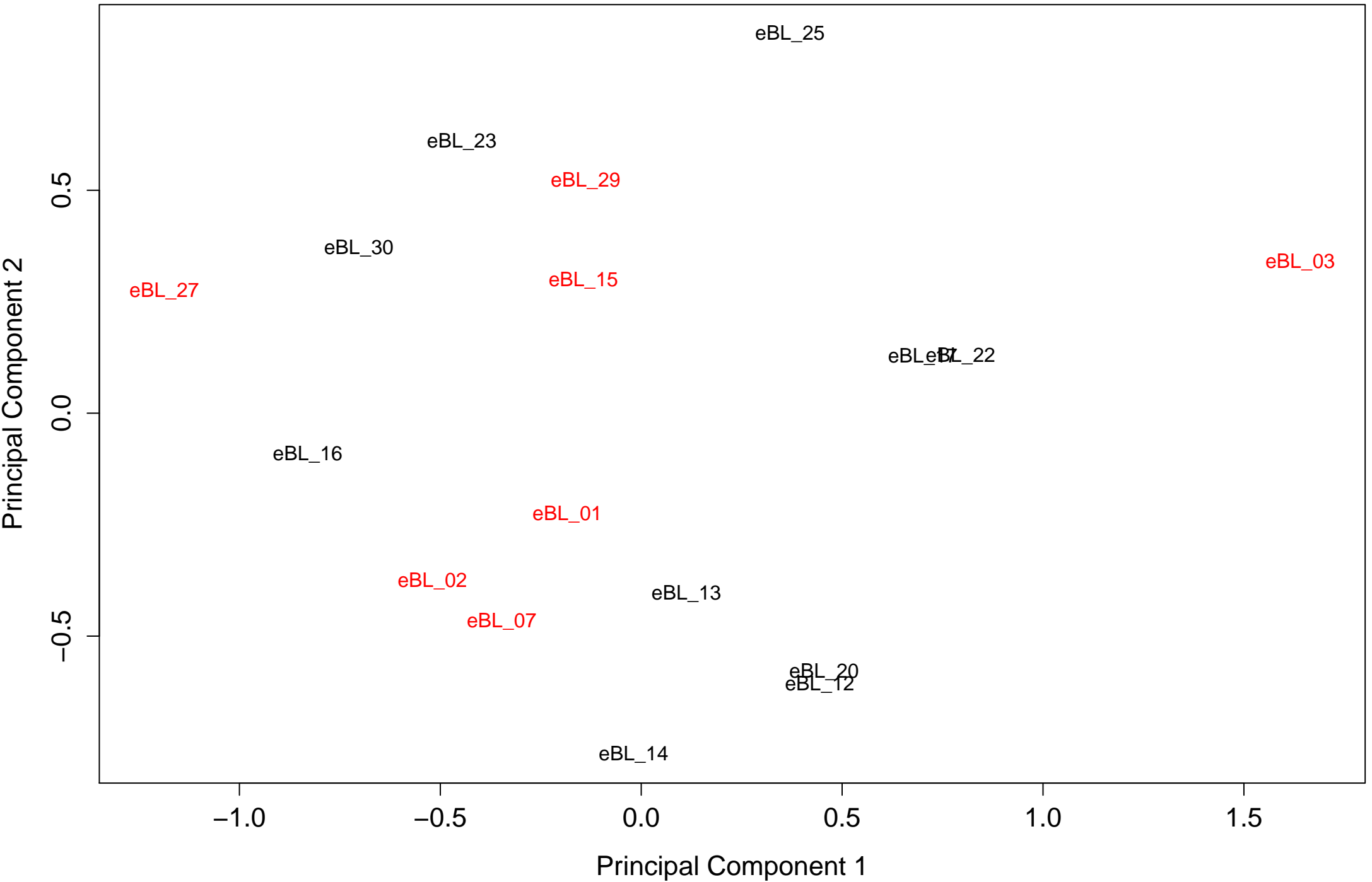

Supplement: Figure S3 — PCA plots of eBL tumors sites. The sample names were colored per their groups (red represents the jaw tumors and black represented the abdominal tumors). The tumor samples don't show well-defined grouping based on tumor site in the PCA analysis. [file Image3.PDF]

Pearson cor: 0.7747127, p-value=0.000425

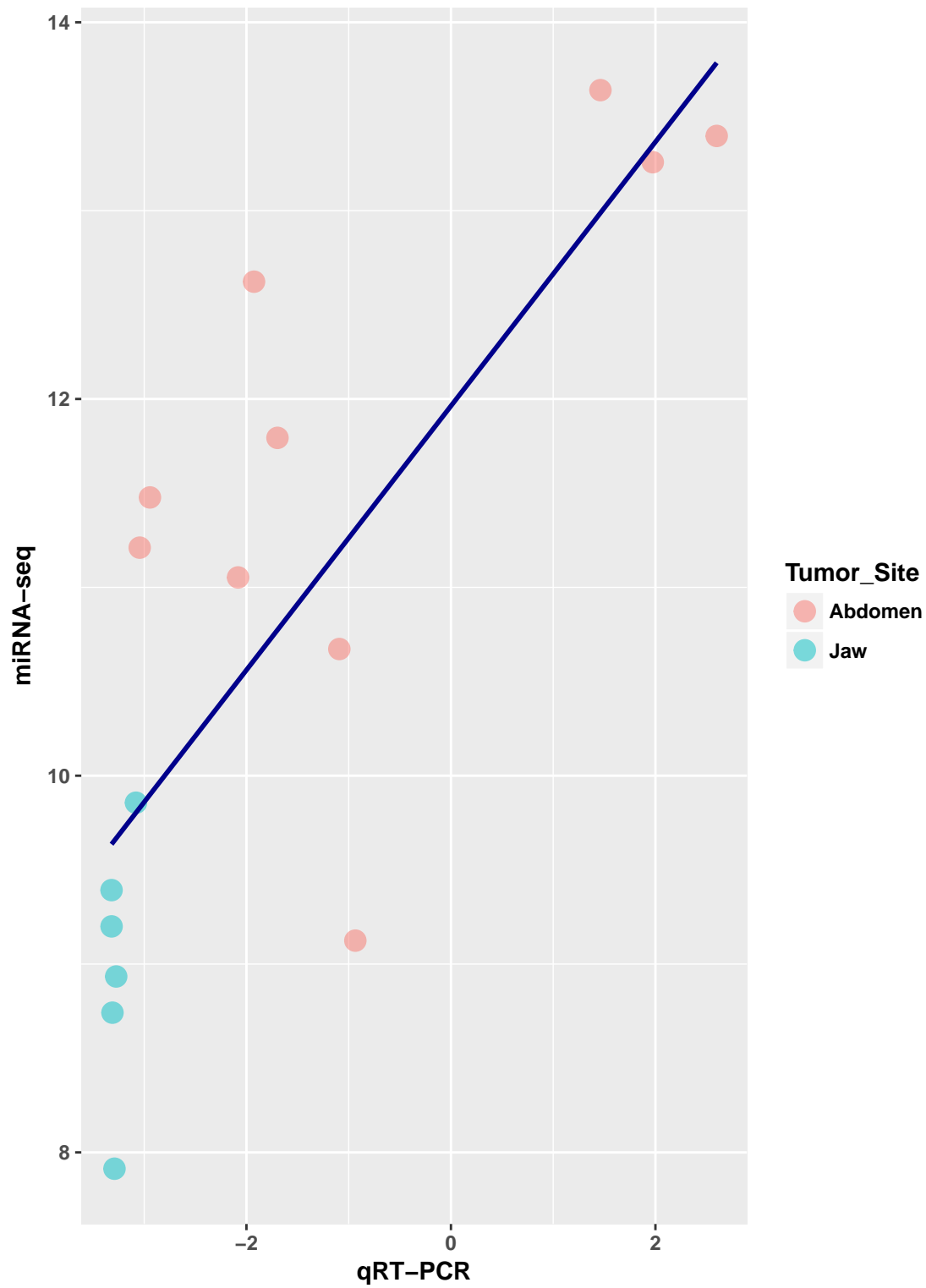

Pearson cor: 0.8189868, p-value=0.00112

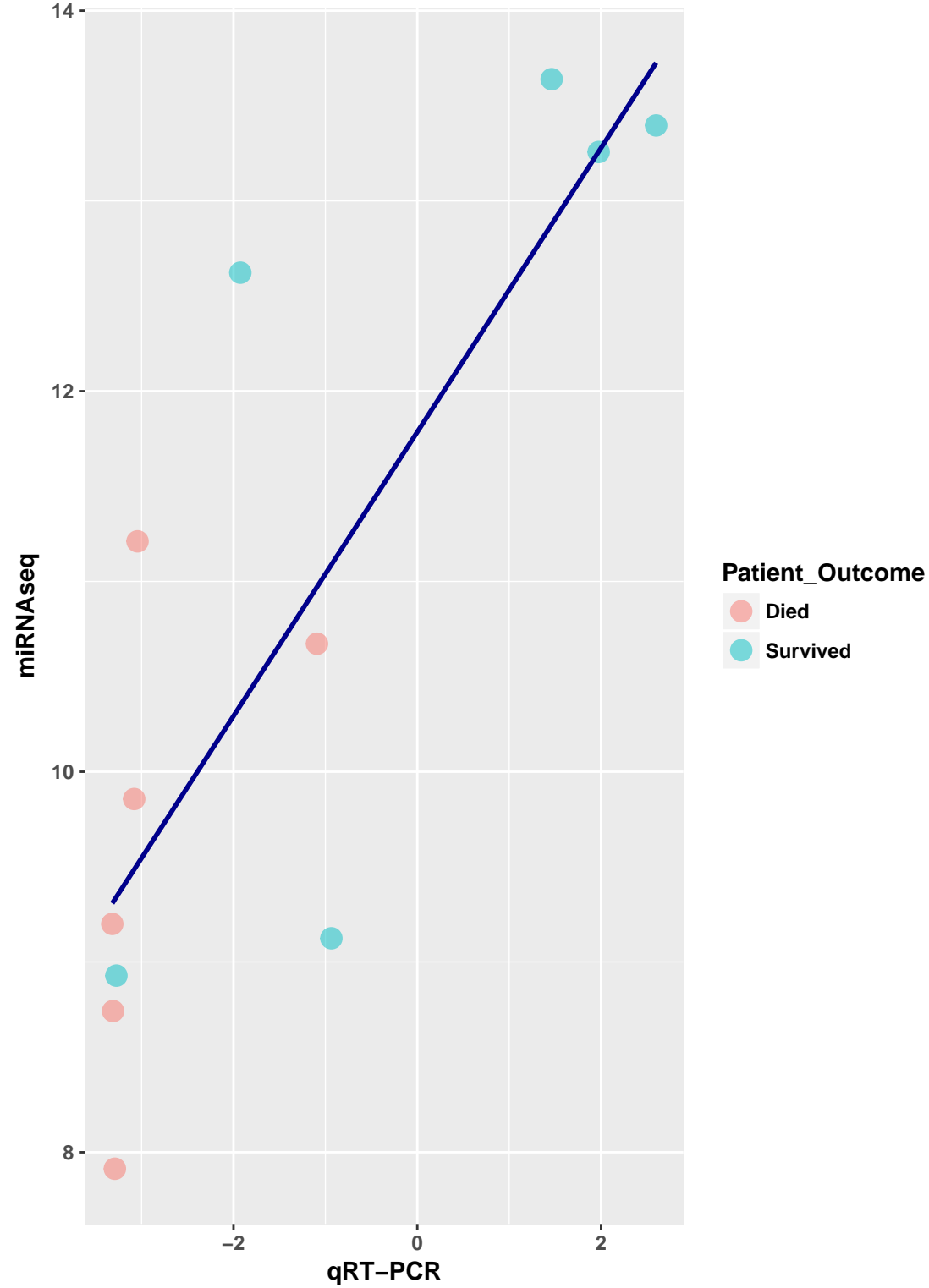

Supplement: Figure S4 — Pearson correlation scatter plots showing a positive correlation between qRT-PCR miR-10a-5p expression levels and miRNA-seq miR-10a-5p expression levels. [file Image4.PDF]

Pearson cor:  $-0.6336$ , p-value= $0.0112$

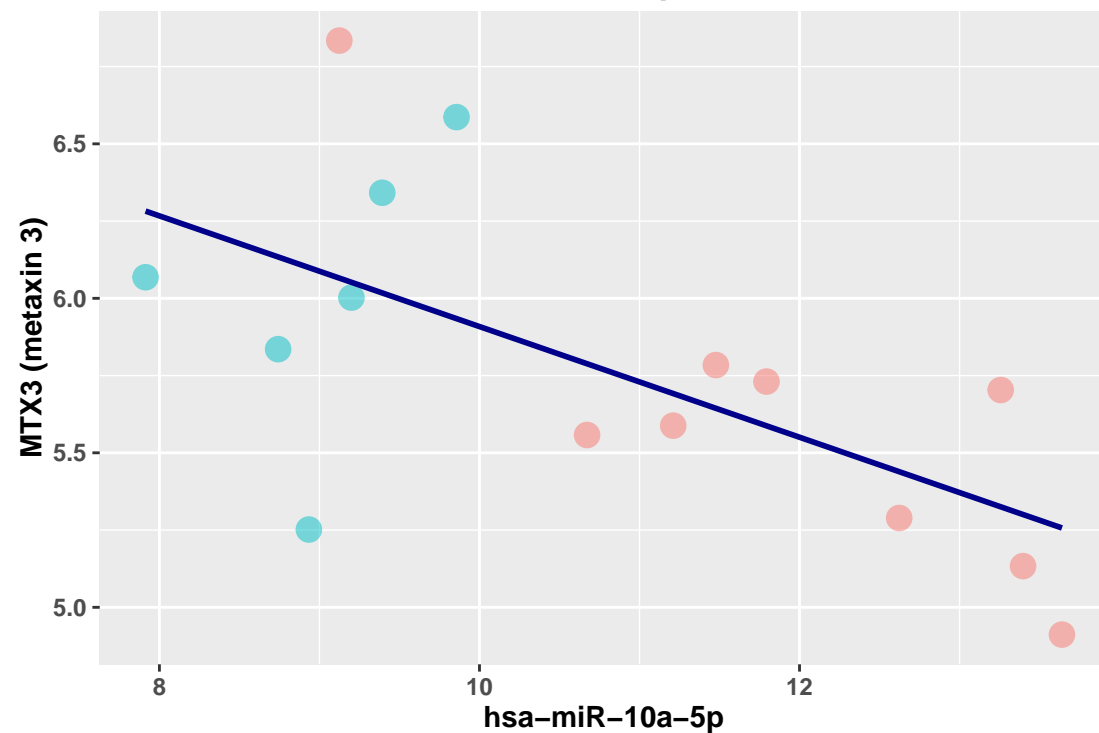

Pearson cor:  $-0.5910$ , p-value= $0.0203$

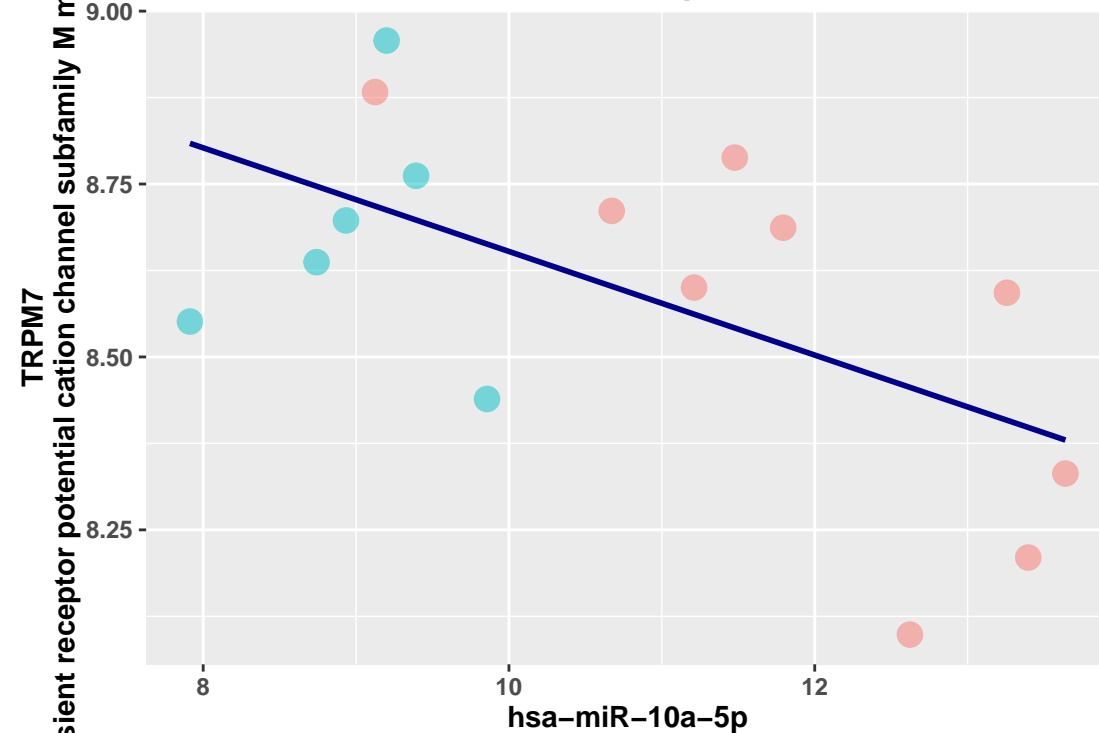

Pearson cor:  $-0.5151$ , p-value= $0.0494$

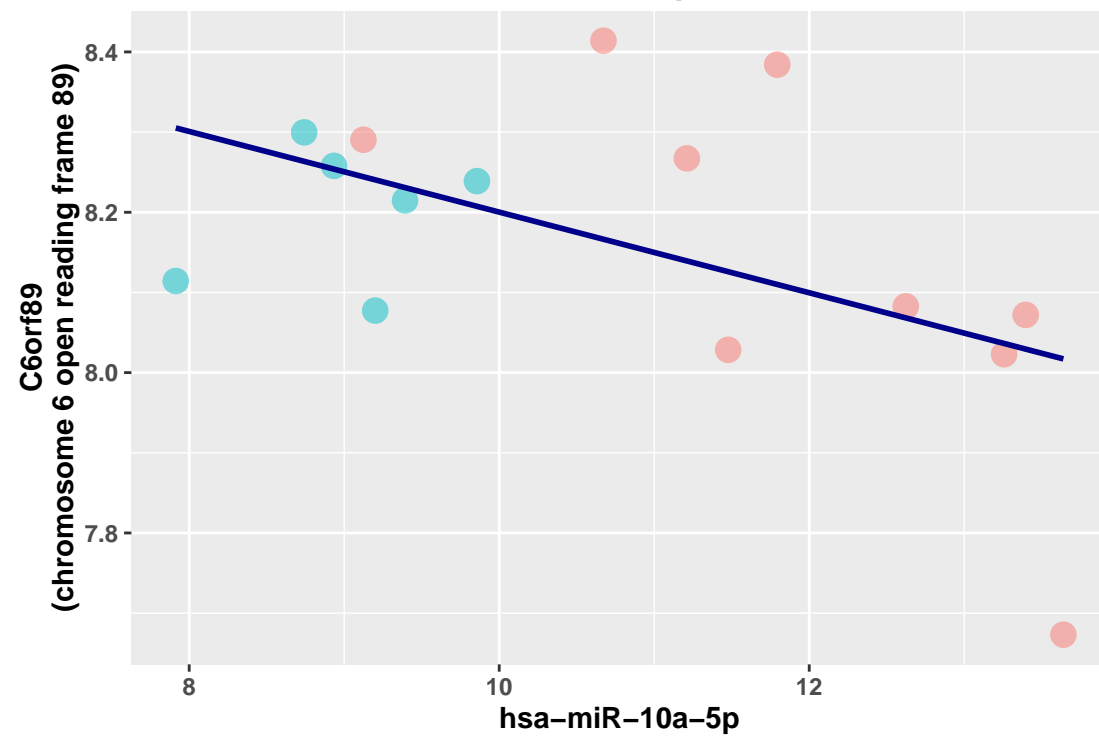

Pearson cor:  $-0.5101$ , p-value= $0.05205$

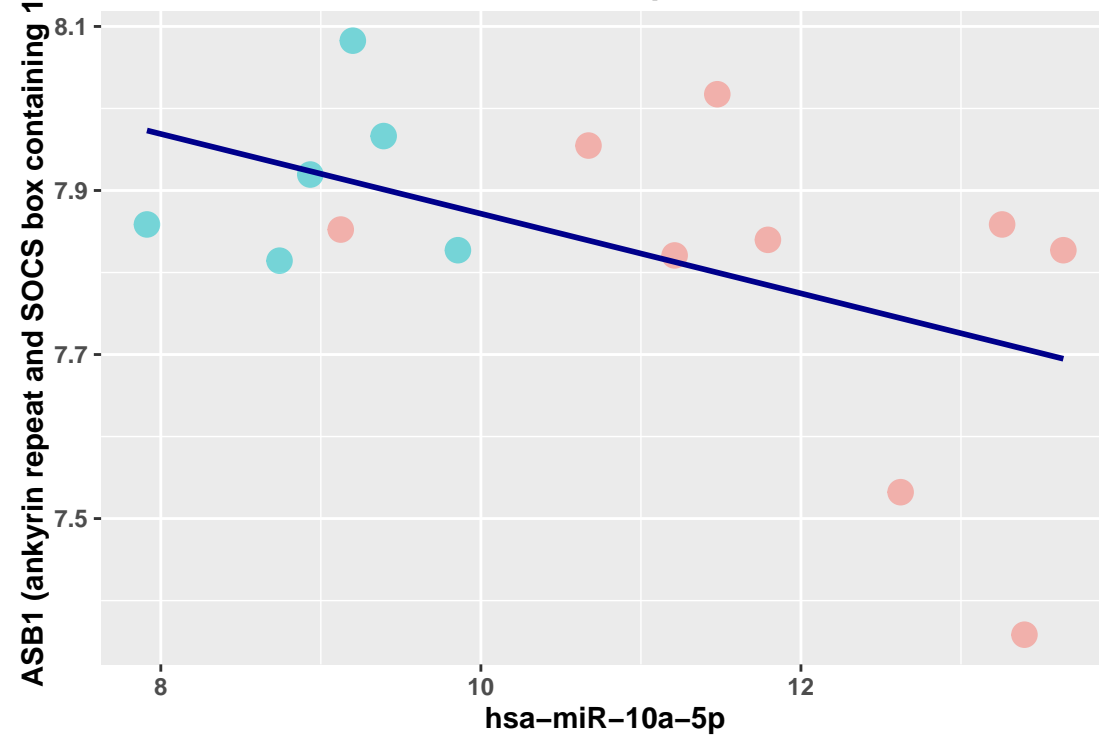

Supplement: Figure S5 — Pearson correlation scatter plots showing the relationship between hsa-miR-10a-5p expression and its target gene expression in eBL patients. The blue points represent the eBL patients with the jaw tumors and the red points represents eBL patients with the abdominal tumors. [file Image5.PDF]

Gene Ontologies enriched  
by the validated target genes  
of hsa-miR-10a-5p

Enriched Gene Ontologies

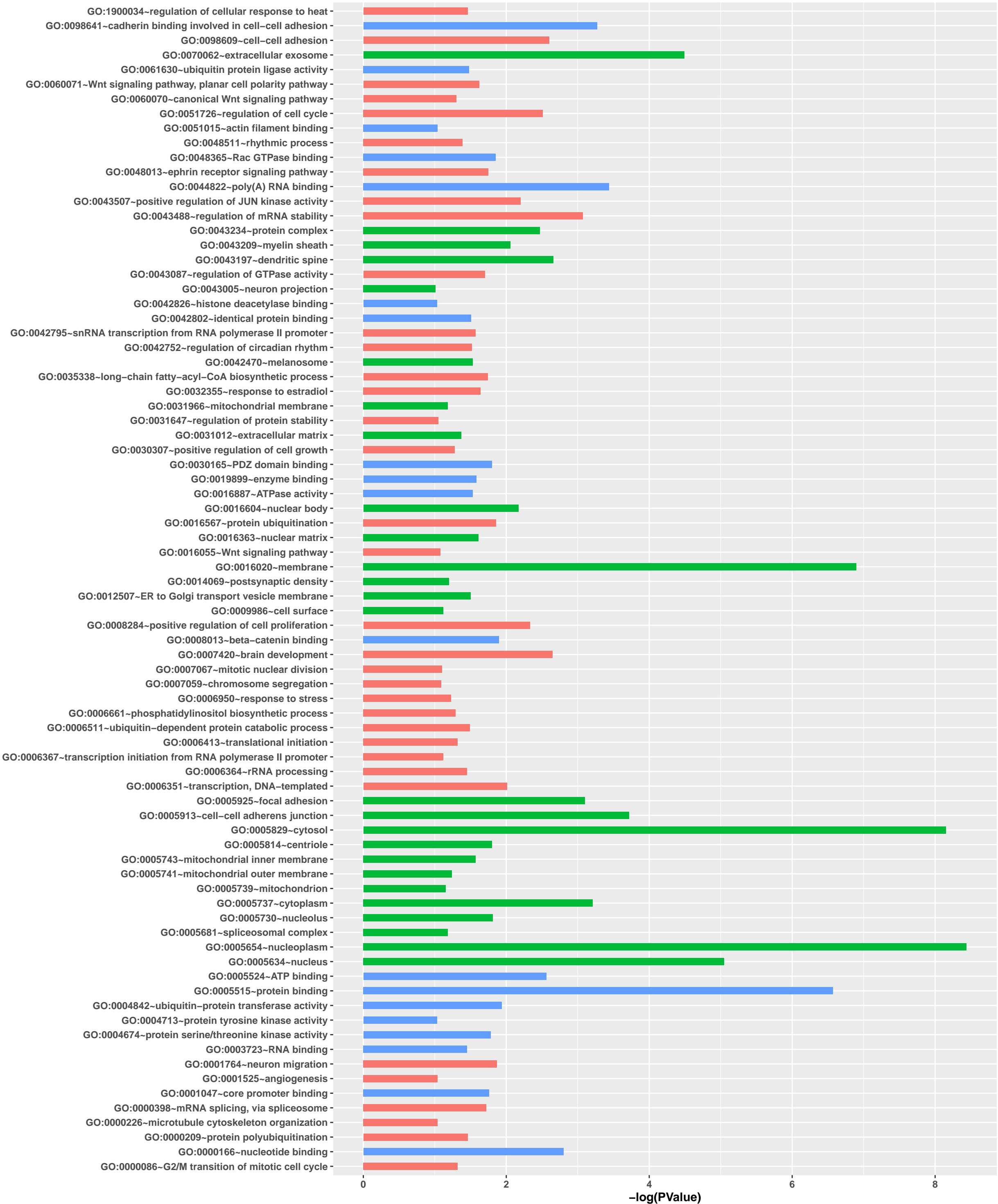

Supplement: Figure S6 — Gene ontologies enriched (Biological processes, Cellular components and Molecular functions) by the validated target genes of miR-10a-5p. [file Image6.PDF]
